# Measuring the Effectiveness of Both Cognitive and Emotional Forms of Instructional Videos Related to the Beef Industry

**DOI:** 10.3390/ani15172584

**Published:** 2025-09-03

**Authors:** Savannah Locke, Karen Hiltbrand, Katie Corbitt, Darcy Richburg, Gabriella Johnson, David Shannon, Soren Rodning, Jason Sawyer, Donald Mulvaney

**Affiliations:** 1 Department of Animal Sciences, Auburn University, Auburn, AL 36849, USA; sll0030@auburn.edu (S.L.); kzh0119@auburn.edu (K.H.); kec0139@auburn.edu (K.C.); gfj0006@auburn.edu (G.J.); rodnisp@auburn.edu (S.R.); jts0109@auburn.edu (J.S.); 2 College of Agriculture, Auburn University, Auburn, AL 36849, USA; dmh0008@auburn.edu; 3 Department of Education, Auburn University, Auburn, AL 36849, USA; shanndm@auburn.edu

**Keywords:** animal welfare, beef, diet/health, environment/sustainability, video

## Abstract

With so many people becoming distanced from the world of agriculture, what is the best way to bridge the knowledge gap? Studies have shown that video messaging could be a key factor in lessening this gap. This study assessed the perceptions of young adults about animal agriculture and the effectiveness of emotional and cognitive videos featuring local farmers and industry experts to alter perception and build trust in the beef industry. An invitation to participate was sent to 10,000 Auburn University students, and responses were closed after 500 complete responses were received. Participants were directed to a Qualtrics (2022) survey with a 5-point Likert scale and open-ended questions. The questionnaire included opinions on animal welfare, the diet/health of red meat consumers, and environmental/sustainability aspects of the beef industry. After viewing one emotional and one cognitive video, each lasting four minutes, participants retook the survey. Data were analyzed using paired *t*-tests in SPSS (Version 28). Results showed participants’ views improved by 82% after watching the videos. ATLAS (Series 9) was used to code key positive and negative words in open responses. Participants reported a stronger preference for the emotional video compared to the cognitive video (190 vs. 99, *p* < 0.05). However, because the videos were always shown in the same order, this finding should be interpreted with caution, as order effects may have influenced participants’ responses.

## Simple Summary

Many people today are unfamiliar with how their food is produced, especially when it comes to raising animals for meat. This lack of understanding has led to confusion, mistrust, and the spread of negative messages about the beef industry. This study looked at how videos could help people learn more and feel more confident about beef production. College students were asked to share their opinions about topics like how animals are treated, whether red meat is healthy, and how beef affects the environment. Then, they watched two short videos—one that focused on emotions and real-life stories and another that explained facts and science. After watching the videos, many students changed their views toward a more positive direction. The emotional video had a stronger impact than the fact-based one. These results show that videos can be a powerful way to help the public understand and trust what happens in the beef industry. Sharing honest stories and clear information can help build support for social licensure for animal agriculture. This kind of communication is important for the future of farming and food production.

## 1. Introduction

Consumers’ purchasing decisions control the market in any industry. According to a market research study, 50 percent of millennials would buy more beef if they knew more about the cuts they were buying, and millennials are expected to continue to impact the products in demand [1,2]. Knowing that purchasers control the demand for products, it is of increasing value that consumers are knowledgeable of where their food comes from and specifically how beef is raised. Millennials lacking knowledge of the beef industry [2] reinforce how important readily available and accurate information is.

Despite modern agriculture’s significance to global food systems and sustainability, there remains a persistent and striking disconnect between public understanding and reality—commonly referred to as the “knowledge gap” in animal agriculture. Consumers often vastly overestimate the role of pasture-based farming and underestimate the prevalence of confinement systems [1,3], while welfare-oriented labels such as “free-range,” “cage-free,” or “humanely raised” evoke expectations of higher animal welfare that are not always met in practice [4,5]. Although surveys indicate strong public concern for animal welfare and expressed interest in higher-welfare options, these attitudes frequently do not translate into purchasing behaviors or policy support [6]. Additionally, there is a widespread lack of consumer awareness regarding routine industry practices and the environmental footprint of livestock production, which remains significant yet poorly recognized in public discourse [7]. Contributing factors include urbanization, inconsistent media coverage, confusing labels, and advocacy messaging from multiple sides—creating a complex information environment that makes informed decision-making difficult [8]. Collectively, these gaps highlight the urgent need for evidence-based communication strategies—such as the comparison of cognitive (scientific) versus emotional (narrative) instructional messaging explored in this study—to more effectively bridge knowledge deficits, rebuild trust, and support informed engagement with animal agriculture.

Presently, consumers source a wealth of information from social media instead of scientific and factual sources. This knowledge gap has been created between the average consumer and the animal agricultural industry, specifically the beef industry, because of where purchasers gain knowledge and the type of media where it is accessed from [2]. Consumers have a tendency to value labels such as “pasture-raised” when determining purchasing decisions [9,10]. Another study came to the conclusion that some consumers will strictly make decisions based on morals and will choose a more “animal-friendly option” [11,12]. Another determinant in the purchase of meat can vary by context, such as if the meat came from a reliable restaurant or grocery store that advertised more humanely treated animals [13]. The public does not want to engage in support of a perceived factory farming framework. They want to make sure their food is raised humanely and safely.

Social media seems to be an excellent way to communicate between different groups of people [9]. Using videos could be an effective tool in order to present educational opportunities for those far removed from the farm to learn about the livestock industry. Randolph and others performed a study that involved using six different videos as a way to communicate the practice of food safety procedures to the public [10]. Three of the videos were analytical in short, medium, and long timings, while the other three were on an easier level to understand [10]. Their finding concluded that people prefer to view videos based on shorter times. Social media could very well play a role in bridging this educational gap. Harnessing the influential power social media presents is a useful form of communication [14]. Media outlets are readily available to help showcase the livestock industry [14]. Media outlets can help diffuse some of the misinformation that is spreading about the industry by activist organizations. Diffusion is a sequential process where something is communicated via specific channels by individuals of a group and then becomes widely accepted as truth or fact [12]. Trust can be established using videos as a communication modality to get a point across. Video has the capability to significantly improve learners’ ability to retain, understand, and transfer new knowledge [15]. This information led to the present study of using cognitive or emotional videos in order to determine if the lack of knowledge between the beef industry and the general public can be improved. A study concluded that people prefer to view videos based on shorter times in an easier way to understand, including everyday language [8]. Across literature findings, the top three concerns of consumers focus on animal welfare, the diet/health of red meat, and the environmental impact of beef cattle.

As a framework, social judgment theory was considered. Social judgment theory is described as key to understanding communication and attitude change while defining the ways possible in which people can alter, judge, and respond to influences on the basis of prior viewpoints [8]. Overall, people will simply choose to believe what they want because they can or have prior feelings about a subject before being properly informed. It was hypothesized that if people were to view the information from the contrasting videos, they would pull from their already formed previous opinions about the beef industry and would adapt new and more positive opinions.

### Research Question and Objectives

Specifically, the research problem is that while the beef industry increasingly uses video-based communication to engage and educate consumers, little is known about the relative effectiveness of different video formats—particularly those emphasizing cognitive (scientific) content versus those employing emotional, narrative-based approaches—in shaping consumer knowledge, perceptions, and engagement. The knowledge gap is that existing literature on consumer decision-making and agricultural communication tends to examine message framing, trust, and perceptions in isolation, often without directly comparing the cognitive and emotional instructional approaches in a controlled, experimentally designed setting. This gap limits the industry’s ability to strategically design communication tools that are both scientifically accurate and emotionally resonant.

The objectives of this research include cognitive and utilitarian goals. The cognitive goal of the study was to examine how different forms of video-based instruction—those emphasizing factual, evidence-based content versus those incorporating narrative and emotional elements—affect viewers’ knowledge acquisition, information retention, and conceptual understanding related to beef production systems.

The utilitarian goal was to evaluate the practical implications of these instructional strategies for the beef industry’s communication and education efforts. By identifying which video formats more effectively improve understanding and engagement among target audiences, our findings can inform the design of outreach, extension, and marketing materials that address both consumer information needs and industry transparency objectives. We asked “Will there be a change in the survey participants’ pretest opinions after watching videos which convey information about the beef community?” The hypothesis for each survey question is as follows: participants’ post-test scores should improve (become more favorable) after viewing the videos. Similarly, the null hypothesis states that participants’ post-test scores will not improve (become more favorable) after viewing the videos.

## 2. Materials and Methods

**Figure 1:**
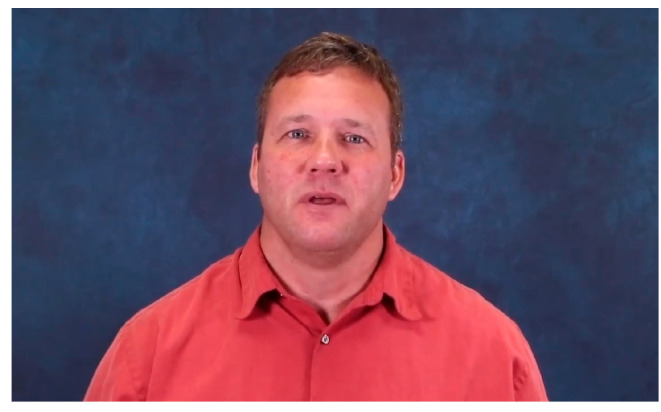
Screenshot of the cognitive video.

**Figure 2:**
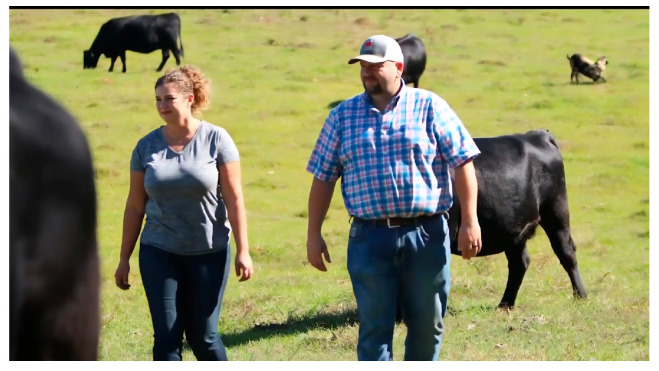
Screenshot of the emotional video.

The purpose of this research was to measure the effectiveness (ability to alter opinions of the beef community) of cognitively based and emotionally based educational videos related to the beef industry in altering pre-viewing perceptions. For this study, two distinct video communication strategies were employed. The cognitively designed video emphasized factual content, logical argumentation, and evidence-based explanations intended to engage participants’ analytical processing. In contrast, the emotionally designed video incorporated narrative framing, imagery, and affect-laden language to elicit empathy and personal connection, thereby engaging affective processing pathways. These design approaches align with dual-process models of persuasion, such as the Elaboration Likelihood Model, where cognitive design reflects central-route (systematic) processing and emotional design reflects peripheral-route (heuristic) processing. The two four-minute videos discussed animal welfare, consumer health, and the environmental impact of beef consumption and production. Videos were produced with the help of local beef ranchers included for the emotional appeal. Recruitment for the ranchers was conducted by consulting with Alabama Cattlemen’s Association (Montgomery, AL, USA) to obtain a list of local producers who were then asked to volunteer. The producers included were all involved in cow/calf operations. Breeds featured in the study were from commercial operations, including Angus, Charolais, Brahman, and Simmental. Interviews were conducted with the three farmers, and recordings of their cattle operations were also used. For a cognitive appeal, the second video was produced by interviewing Dr. Soren Rodning, a State Extension Veterinarian and coordinator of the Beef Quality Assurance certification program (Centennial, CO, USA) for Alabama. Screenshots of example frames from each video are displayed in Figure 1 and Figure 2.

### 2.1. Participant Population

The target population was 10,000 students (undergraduate and graduate), all above the age of 19 (83% were born between the years 1999–2003), 38.4% male/60.3% female, who at the time of the study were currently enrolled in college classes. Participants declared their upbringing environment: 74.3% reported urban and 42.2% rural backgrounds. Participants represented a wide variety of academic disciplines. The largest groups were from the College of Education (19.6%), College of Architecture, Design, and Construction (16.4%), and College of Human Sciences (14.8%). Other represented colleges included the College of Business (7.8%), College of Pharmacy (11.2%), College of Agriculture (5.5%), College of Liberal Arts (5.0%), College of Forestry and Wildlife Sciences (4.1%), College of Engineering (2.7%), College of Nursing (0.2%), and College of Sciences and Mathematics (0.2%). Based on program curricula, the majority of participants were not expected to have extensive prior exposure to animal production systems or beef cattle production, either through coursework or practical experience. The target population was not an at-risk population and did not endure any negative consequences from completing the survey. Participation was voluntary, and respondents could exit the survey at any time, and their responses were both anonymous and unidentifiable. Any incomplete surveys (including those exited before completion) were deleted during the data cleaning phase and not used. No face-to-face interactions occurred; the consent process and all study procedures were completed online. The survey was approved by the Institutional Review Board (IRB) 21-141. The latter is a committee that reviews research proposals involving human subjects to ensure the research is ethical and protects the rights and welfare of participants. IRBs assess the potential risks and benefits of research, ensure informed consent is obtained, and monitor studies throughout their duration.

Surveys were administered to all participants online through Qualtrics. Upon completion of demographics and the pre-test questions, participants viewed two videos. Both videos contained scripted content related to the agriculture/beef industry. One video was centered around appealing to viewers’ emotions and contained minimal facts or figures. The other video focused on appealing to cognitive responses and included statements on referenced facts, figures and statistics related to agricultural science as well as the beef industry. A post-survey was then administered to evaluate the overall impact of both videos to reveal any potential differences in effectiveness regarding the emotional vs. cognitive-based videos and assess whether consumer perceptions changed, becoming more positive or more negative toward the beef industry.

### 2.2. Recruitment Process

The Office of Institutional Research at Auburn University forwarded the motivational email across campus, inviting students for voluntary participation. Upon receiving an invitation, students voluntarily completed a survey that polled demographic information such as gender, age, diet, knowledge of agriculture, and political affiliation, as well as if they are involved with agriculture. The survey followed up with questions about whether they purchase beef or beef-derived products, what affects their decision-making and fact-based questions about the agriculture/beef industry. It is important to note that students who do not purchase beef or beef-derived products could still participate if interested. The only exclusions from the data collection were those who did not fully complete the survey.

### 2.3. Statistical Analysis and Instrumentation

In addition to soliciting demographic information, a pre-viewing survey and a post-viewing survey were administered in Qualtrics (2022). SPSS (Version 28) was used for frequency and analysis of quantitative data derived from a Likert scale. A combination of paired-samples *t*-tests and descriptive statistics was used to determine results from the pretest, and the posttest was used. The post-survey results evaluate the overall impact of both videos to reveal any potential differences in effectiveness regarding the emotional vs. cognitive-based video as well as to see if the consumer’s perceptions changed, whether it be more positive or more negatively geared towards the beef industry. ATLAS (Series 9), a qualitative data collection software used for qualitative data analysis, was utilized for thematic coding of responses to open-ended questions. Five questions related to each of 3 main topics—animal welfare, diet/health of red meat, and environment/sustainability—were asked. A five-point Likert-type scale was used with the response categories: strongly agree (1); somewhat agree (2); neutral (3); somewhat disagree (4); and strongly disagree (5). In addition to the fifteen questions, participants were asked which video they preferred, in what ways the videos watched could be improved, which aspects of the videos most influenced their opinions about the beef industry, whether they had ever viewed anything similar to what they saw in the videos, and, after viewing the videos, whether they had a more of a positive or negative view of beef cattle production

In comparing the responses from the pretest and those collected during the posttest, equal variances were not assumed. Statistical significance of calculated scores was measured using a statistical significance of *p* ≤ 0.05. Paired *t*-tests were used to determine changes in item means from the pre- and post-survey responses. Qualitative responses for the questions, “In what ways could the videos you viewed be improved?” and the question, “After viewing the videos, do you have more of a positive or negative view on beef cattle production?” were analyzed for themes.

## 3. Results

The pre-survey and post-viewing survey yielded varying perception results. Due to the nature of the questions framed in a positive or negative tone, each statement is discussed in relation to its significance level calculated. More specifically, the participants recorded strong perception changes after viewing the videos.

Viewing the videos elicited a positive attitude alteration regarding the animal welfare practice statements. Animal welfare statements yielded significant (*p* < 0.001) positive change in perception; therefore, participants responded more positively to statements related to animal welfare after watching the videos. This suggests that after viewing the videos, the participants had a more favorable perception of farmers treating their beef animals humanely, respectfully, and in a way that meets current welfare standards (Table 1). Also, after viewing the videos, it is suggested that participants understand that animals should be treated in sickness through means of rest, antibiotics, or medicine (Table 1). Four of the five statement responses were more positive in the post. The fourth statement, “I believe beef cattle deserve to have access to clean water, fresh grass, and healthy feed” had a higher mean on the post-viewing survey so people were more likely to disagree. All 5 items reached significance toward a more positive view. That is, after viewing the videos, participants were more likely to express positive beliefs related to the welfare of cattle.

In regard to diet and the health of beef, participants demonstrated a split in decisions through their responses. Four of the five changes were statistically significant (*p* ≤ 0.05). Three of the four shifted in a more positive direction in favor of the beef industry. The data suggests that after watching the emotional and cognitive videos, participants will continue purchasing beef products (*p* < 0.001), perceive beef as safe to consume (*p* = 0.035), and believe red meat is healthier than plant-based proteins (*p* < 0.001) (Table 2). While the results suggest that the videos did not shift participants’ perception of support for the sale of beef products and that their opinions remained stable after watching the videos, both pre- and post-viewing values averaged 1.5, which is indicative of high support for the sale or purchase of beef.

Respondents had mixed changes (*p* < 0.05) in perception regarding the environmental area of our research or the sustainability of producing beef. All 5 questions shifted significantly. However, 3 out of 5 shifted positively. After viewing the videos, participants were significantly more likely to agree that farmers should communicate with the public and less likely to agree that farmers do not care about the environment and that the beef cattle industry should be phased out. On the other hand, participants were more likely to agree that farmers are the main contributors to pollution and believe that the beef cattle industry is not sustainable (Table 3).

The results from the paired samples *t*-test provided us with insights on perception shifts from participants after watching the videos. Since the results showed the videos producing both positive and negative shifts in perception, these data provided us with an understanding of what is best communicated through videos and perhaps where the industry can improve in communicating topics about the beef industry. Specifically, animal welfare practices are communicated effectively, while some aspects of the diet and health of beef products and especially the environmental aspects of the beef industry could be improved in order to influence positive shifts in opinion.

Considering whether video communication could be effective or not, we looked at its impact through a couple of free-response questions. For example, we wanted to see if anyone had observed how effective the messaging could be (Table 4). Due to such positive responses, video would indeed be an excellent tool for teaching and engaging future audiences.

Overall, we wanted to examine how effective the videos were in changing people’s perceptions of the beef industry. Out of the 313 respondents, 74.4% had a more positive response (Table 5). The initial hypothesis was proven correct in the fact that a majority of responders were left with a positive view after completing the surveys. Respondents also preferred the emotional video over the cognitive video (Table 6).

Responses to the question, “In what ways could the videos you viewed be improved?” were coded as positive, negative, or neutral for emerging themes. As far as positive themes, the top three common codes were that “Farmers take good care of their animals” (1) “Beef is healthy and an essential part of our diet” (2) and “I gained knowledge and a new perspective of the beef industry” (3). When asked about negative factors, the top three common responses were, “Beef is environmentally unsustainable” (1), “Beef is inhumanely harvested” (2) and “All beef is factory farmed” (3).

Emerging themes in response to the question, “Which aspects of the videos really influenced your opinions about the beef industry?”, 82% were positive, 11% were neutral, and 7% were negative. As far as the top three positive themes, the themes were “The videos provided a transparent view about the beef industry” (1), “food animals are raised wholesomely, respectfully, and humanely” (2) and “the videos influenced me to have a more positive outlook on beef” (3). The top three negative themes were “my opinions did not change” (1), “the information provided was staged and biased” (2), and “more facts and statistics would help explain beef production” (3).

## 4. Discussion

With the increasing popularity of virtual messaging, there is a need for a more transparent view of the beef industry. Videos are proposed as an effective tool to communicate about specific topics, and there is a great opportunity for animal agriculture to implement them to increase transparency, communicate to a broader audience, and bridge the knowledge gap between consumers and producers. In this study, participants were shown two separate videos pertaining to emotional and cognitive aspects related to the beef industry. The main focal points are on the aspect of animal welfare, the diet/health of red meat, and environment/sustainability, which were explored through several studies. This study is similar to studies that provide a visual experience [10,16]. This present study focused on providing emotional and cognitive videos as tools in order to engage participants. Crafted by a panel of animal science experts, each video created two different narratives for the participants to connect with. The emotionally charged video had a combination and overlap of stories from actual Alabama beef cattle producers. The producers, all from the same family, shared their experiences and hardships, as well as their motivations to farm beef cattle to create an atmosphere of family values and realism tied to the industry. It was anticipated that these emotionally charged characteristics would be more influential on participants’ perceptions toward beef production. The cognitively charged video portrayed facts and statistics about the beef industry and the quality of beef products communicated by an actual practicing extension veterinarian. Dr. Soren Rodning presented the same information as the emotional video in an academic or educational perspective. Expert scripts were prepared for both videos; however, the spontaneous, unscripted content proved more engaging and was therefore chosen for the projected participants.

In essence, the application of Social Judgment Theory is utilized in this work, as persuasion is not just about presenting information but also about understanding or considering an audience’s existing framework of beliefs and how new information may be perceived in relation to it. In designing advertising, informative, or persuasive communication, an understanding of Social Judgment Theory may help science communicators tailor their messages to be more effective by considering the existing attitudes and beliefs of their audience. For example, an advertisement about animal agriculture might be more effective if it subtly introduces cognitive information or visual benefits that are consistent with existing consumer preferences, rather than directly contradicting them.

Consistent with Social Judgment Theory [4,8], it was hypothesized that the intervention of videos will shift opinions optimistically in a positive outlook, especially the emotionally charged video regarding the beef industry, and based on the resultant data, it is suggested this is true. In each question subset—animal welfare, diet and health of beef, and environment and sustainability of beef production—significant differences in opinion were recorded post-video intervention. Overall, animal welfare topics demonstrated the highest potential to shift opinion. Results from the diet and health of beef and sustainability of beef production sections were less conclusive than the animal welfare portion, but the results still suggest that video messaging can be an effective tool to explore its use in the future. This study found that people genuinely liked seeing farmers interact with their cattle and gained knowledge. In contrast to the study by Ventura [16], focused on bringing people to an actual dairy, the present study showed participants the speakers’ real-life farms.

Video messaging, growing in popularity, has immense potential to alter attitudes toward agricultural topics out of non-agricultural audiences, as shown in this study. Both the descriptive statistics and paired samples *t*-tests results demonstrate this phenomenon. This study found that perceptions regarding animal welfare differed significantly after viewing the videos. Participants perceived the beef industry as a humane, ethical, and safe industry with the specific understanding that beef cattle are kept to current animal welfare standards. Regarding diet, health, and consumption of beef, participants showed significant shifts in understanding the health benefits of beef. Specifically, participants demonstrated a shift in perception in their confidence that red meat products are healthier than plant-based alternatives. Sustainability of beef, however, demonstrated the least clarity in shifting perceptions of participants. After watching the videos, there were statistically significant perception changes in a negative manner, such as farmers are responsible for current pollution outputs. Considering all of the quantitative measures, qualitative analysis provided greater insight into participant perceptions across the three research areas. Particularly, thematic coding of the open-ended question responses revealed the percentages of both positive and negative comments that addressed the beef industry after participants viewed the videos. For example, the videos provided a generally appreciated level of transparency of the beef industry but were also scrutinized because the farms presented were not “representative of factory farms.” Though negative statements like these were commented throughout 405 responses, the videos induced positive outlooks for the beef industry. Thus, reinforcing the concept that video messaging can be an effective tool for promoters of the industry.

Animal welfare seems to be the biggest concern among college students in this present study, as in several others [3,6,8,14]. As far as study results go, consumers’ strongest positive comments were about the fact that beef cattle were being treated humanely by their owners. The most frequent concern was the well-being/welfare of the cattle, followed by environmental impact [4]. Unlike the results in Ventura et al. [16], after seeing the videos, the participants’ behaviors in this study generally improved in their attitudes towards animal welfare, diet/health of red meat, and environment/sustainability. It seems to be more effective to show videos than to bring consumers to actual locations.

### Limitations and Further Research Discussions

It is acknowledged that there is substantial diversity of animal farming constructs, and additional research should examine the impact of these different settings in different video options. If given the chance to redo this study, the formatting of the questions in Qualtrics would be changed to remain neutral and consistent in design for all questions. For future research, the study could be conducted across multiple colleges and universities to compare results. There could be more diversity due to the fact that mostly Caucasian men and women were shown. Analysis of demographics could be added alongside the pretest and the posttest to create insights into influences on data.

Gender: At 65.6% female and 33.4% male, the study may be influenced by gender differences. Men and women often differ in meat attitudes and message receptivity. Men typically report higher meat consumption and associations of meat with strength and masculinity, whereas women more frequently express concerns about animal welfare and the environment—and may be more open to emotional messaging [17,18].

Age/Cohort: Younger cohorts (Millennials and Gen Z, as represented in this study by design, were 75% of participants) demonstrate greater concern for sustainability, animal welfare, and alternative proteins. They may respond more to value-driven or narrative messaging, even while they can be skeptical of purely factual appeals [18].

Our participants represented most majors at the university. It is acknowledged that students studying animal or agricultural sciences generally have higher subject familiarity and more stable attitudes. These participants may show smaller pre–post knowledge gains but deeper engagement with technical content, while non-ag students may exhibit larger shifts from both cognitive and emotional formats [19,20]. Yet only 15% of the participants were in agriculture.

Political Ideology: In this study the demographics of participants were 52.9%, 15.9%, and 11.1% for Republican, Democrats, and independents, respectively. Political affiliation influences how individuals process scientific and environmental messages. Ideologically congruent frames (e.g., economic stewardship vs. welfare) enhance persuasion; incongruent frames can trigger motivated reasoning and rejection of the message [21].

Rural vs. Urban Background: While the percentages of rural and city were 42 and 47%, individuals raised in rural or agricultural environments often have greater familiarity with farming practices and may be less persuaded by generalized messages than urban participants, who frequently have larger baseline knowledge gaps [22].

Agricultural Experience: Twenty-three percent of the participants declared agricultural work experience, while 76.8% had none. This is important, as prior exposure to farming or agricultural work usually yields higher factual knowledge and entrenched beliefs. Such individuals may benefit more from factual (cognitive) messaging, while narrative approaches may be more effective in reaching lay audiences [23].

Meat Consumption Habits: This was not a declared pre-survey demographic, but meat purchasing was included in the pre- and post-survey. It is noteworthy that individuals with strong meat attachment are more likely to resist messages framed around production risks or welfare concerns, whereas occasional consumers may be more open to persuasive framing [17,18].

Furthermore, if the videos were launched on more social media platforms, they might lead to a more realistic and transparent view of the beef industry, helping to develop a sense of trust between the general public and the beef industry.

## 5. Conclusions

There is a need for a more transparent view of the beef industry as more people are becoming increasingly removed from the farm. Fewer people can identify where the meat comes from and have been exposed to social media narratives and messaging about inhumane treatment, diet-health consequences and relationships to environmental sustainability. By using digital media such as video, a climate of openness can be created, which can be utilized to bridge the gap in understanding between the general public and the beef industry.

This study was extremely successful in showing that people’s perceptions can be changed for the betterment of the industry. Videos are an excellent form of communication and need to be explored as a communication modality to lessen the knowledge gap between citizens and the beef industry. The results of this study show that people have an interest in learning more about the beef industry. They also need more facts and more open communication regarding the concepts of diet/health and environment/sustainability.

## Figures and Tables

**Table 1:** Paired sample *t*-test statistics for participants’ responses regarding animal welfare ^1,2,3,4^.

Pair of Pre and Post	Mean Pre	Mean Post	*T*	*p*
I believe beef cattle are humanely treated.	2.78	1.61	19.556	<0.001
I believe that it is necessary to treat sick animals. Such treatments could include rest, antibiotics, or medicine.	1.40	1.22	5.228	<0.001
I think farmers treat their beef cattle with respect.	2.33	1.56	13.977	<0.001
I believe beef cattle deserve to have access to clean water, fresh grass, and healthy feed.	1.29	1.44	−3.381	<0.001
I believe farmers treat animals in a way that meets current animal welfare standards.	2.37	1.53	15.499	<0.001

^1^ Survey of young adult college students about their opinion of the welfare of animals prior to and after the viewing of cognitive- and emotionally based videos. N = 326. ^2^ Mean after viewing the videos. ^3^ Results created using a *t*-test from SPSS. ^4^ A five-point Likert type scale was used with the response categories: strongly agree (1); somewhat agree (2); neutral (3); somewhat disagree (4); and strongly disagree (5).

**Table 2:** Paired sample statistics for diet/health ^1,2,3^.

Pair of Pre and Post	Mean Pre	Mean Post	*T*	*p*
I purchase beef products weekly.	2.54	1.46	16.251	<0.001
I believe that beef cattle should not be consumed.	4.42	4.29	2.473	0.014
I support the sale of beef products.	1.56	1.58	−4.09	0.683
I believe that red meat is healthier than plant-based proteins.	3.40	2.32	9.377	<0.001
I believe that beef is safe to consume.	3.40	2.32	2.120	0.035

^1^ Survey of young adult college students about their opinion of the diet/health of animals prior to and after the viewing of cognitive- and emotionally based videos (N = 326). ^2^ Mean after viewing the videos. ^3^ A five-point Likert-type scale was used with the response categories: strongly agree (1); somewhat agree (2); neutral (3); somewhat disagree (4); and strongly disagree (5).

**Table 3:** Paired samples statistics for environment/sustainability ^1,2,3^.

Pair of Pre and Post	Mean Pre	Mean Post	*T*	*p*
I believe that farmers do not care about the environment.	4.21	4.43	−3.949	<0.001
I believe farmers are the main contributors to pollution.	4.30	2.24	19.823	<0.001
I believe the beef cattle industry is not sustainable.	3.55	1.95	13.744	<0.001
I believe animal agriculture is a large contributor to pollution and should be phased out.	4.06	4.24	−3.782	<0.001
I believe that farmers should communicate with the general public about their farming practices.	2.01	1.68	6.165	<0.001

^1^ Survey of young adult college students about their opinion of the welfare of animals prior to and after the viewing of a cognitive- and emotionally based videos (N = 326). ^2^ Mean after viewing the videos. ^3^ A five-point Likert-type scale was used with the response categories: strongly agree (1); somewhat agree (2); neutral (3); somewhat disagree (4); and strongly disagree (5).

**Table 4:** Frequency responses to the question, “Have you viewed anything similar to what you were shown today?” ^1,2^.

Response	Frequency	Percent
Yes	132	42.2
No	181	57.8
Total	313	100

^1^ Survey of young adult college students on perceptions about beef animal topics after viewing a cognitive- and an emotionally based video. ^2^ Results created using a frequency from SPSS.

**Table 5:** Frequency response to the question, “After viewing these videos, do you have more of a positive or negative view on the beef industry?” ^1,2^.

Response	Frequency	Percent
Positive	233	74.4
Negative	6	1.9
Neutral	74	23.6
Total	313	100

^1^ Survey of young adult college students on perceptions of beef animal topics after viewing a cognitive- and an emotionally based video. ^2^ Results created using a frequency from SPSS.

**Table 6:** Response to the question, “Which video did you prefer? Video 1 or Video 2?” ^1,2^.

Video	Frequency
Video 1: Emotional	190
Video 2: Cognitive	101
Both	14
NA	10
Total	326

^1^ Survey of young adult college students on perceptions of beef animal topics after viewing a cognitive- and an emotionally based video. ^2^ Results created using a frequency from SPSS.

## Data Availability

Data presented in the study are available upon request to the corresponding author.
